# Roadmap for Creating Effective Communication Tools to Improve Health Equity for Persons With Intellectual and Developmental Disabilities

**DOI:** 10.3389/frhs.2022.859008

**Published:** 2022-06-09

**Authors:** Priyanka R. Dharampuriya, Susan L. Abend

**Affiliations:** ^1^Lincoln Memorial University - DeBusk College of Osteopathic Medicine, Knoxville, TN, United States; ^2^The Right Care Now Project, Inc., Westborough, MA, United States

**Keywords:** developmental disabilities, intellectual disabilities, healthcare disparities, health equity, health passports, health information transfer, person-centered care, health assessment

## Abstract

Persons with intellectual and developmental disabilities (IDD) live 20 fewer years than the average person and almost 40% of their deaths are from preventable causes. They suffer from well-documented disparities in health and healthcare, and much of this inequity is rooted in information transfer failures between patients, their caregivers, and their healthcare providers. Tools to improve communication between these stakeholders, such as health checks and hand-held health records, or health passports, have been implemented in Europe, Australia and Canada with mixed results, and there are no standard information tools currently in widespread use in the U.S. We review the evidence of the effectiveness of these tools, as well as their barriers to adoption, to inform proposed development of next-generation information transfer tools most useful to patients with IDD and their healthcare providers. The repair of health information transfer failures will be a major step toward achieving health equity for this population.

## Introduction

Persons with intellectual and developmental disabilities (IDD) experience well-documented inequities in healthcare and marked disparities in health compared to the general population (1). IDD, a condition characterized by significant limitations in both intellectual functioning and adaptive behavior (2) affects <3% of the population, yet has been identified as the third highest risk factor for COVID-related death in the U.S (3, 4). Those with IDD have been found to live 20 fewer years than the average person, with up to 40% of their deaths attributable to preventable causes. Persons with IDD are twice as likely to have unmet health needs (5), have among the lowest rates of receiving preventative care services (6), and frequently suffer missed diagnoses that lead to incorrect management (7). Adults with IDD suffer higher rates of obesity, dyslipidemia, diabetes, low bone density, and osteoporosis (8–11) than the neurotypical population. The healthcare disparities are so great in the U.S. that the American Medical Association has recommended that persons with IDD be designated as a Medically Underserved Population (12).

Research in the U.S and internationally has consistently identified two key reasons for health and healthcare deficiencies in this population, and both are related to failures of information transfer. First, providers often report that they have difficulty communicating with patients with IDD, so are challenged to assess their needs (13–15). Second, multiple studies indicate that over 90% of primary care providers, psychiatrists, and nurses report feeling that they do not have the skills and information to provide proper care for this population (13, 16, 17). While specialized clinics have been funded in a number of U.S. states to address the health needs of the IDD community, the average clinician lacks the expertise to assess and manage the common morbidities this population experiences, such as frequent pneumonia, severe constipation, or abuse. Furthermore, there is no standard way to assure that the average clinician has access to accurate health history information at the point-of-care to do a proper assessment. Therefore, a person with IDD cannot count on getting expert adult care unless they can travel and get an appointment in a specialty clinic.

Communication tools such as “health checks” and “health passports,” first developed in Australia and the United Kingdom, have been used to try to remedy the information failures that prevent persons with ID from obtaining expert care in general healthcare settings. However, none of these have gained widespread acceptance. Based on the implementation experience and effectiveness of the current information transfer tools, we propose a framework for future tool development that will optimize both information transfer and user adoption, and therefore improve healthcare quality and equity for those with IDD.

### Health Checks

In the late 1990s, after recognizing that many conditions affecting the health of persons with IDD are often missed (18), doctors in New Zealand, the United Kingdom, and Australia began developing health assessment checklists to assure general practitioners noted and fully addressed health issues common in the IDD population (19, 20). These assessment checklists were developed for annual use, and vary in length and content. For example, the Comprehensive Health Assessment Program, or CHAP (21), is a 20-page document consisting of health questions filled out by a caregiver, which contains system category areas for the primary care provider (PCP) to fill in their assessments. It also provides the PCP with a general list of problems common in those with intellectual disabilities, along with a list of health issues that present commonly in those with specific genetic syndromes such as Down Syndrome or Prader-Willi Syndrome.

Other standardized health checks, such the Cardiff or Welsh Healthcheck (22), consist of a shorter, targeted checklist of health history, examination, and an action plan section for the PCP to use at an annual assessment. There are now more than 20 available health checks for individuals with IDD, and all vary in both content and workflow. The Scottish 21^st^ Century Check (23) relies on specialized nurses to perform the evaluation and can take up to 4 h to complete. Most recently, the Developmental Disabilities Primary Care Program in Ontario, Canada created an annual checklist tool based on consensus guidelines they developed for the primary care of those with IDD. The tool is a comprehensive checklist for gathering history and includes a template for complete assessment. The developers recommend that the health check be performed over a series of visits (24).

There is good evidence that implementing a health check tool improves care for those with IDD. In one Australian study, 60% more health issues were detected in the patients who had health checks (25), and multiple studies have indicated that health checks resulted in a higher uptake of preventative care (26). In addition, the use of health checks in the UK improved the documentation of chronic mobility and sensory issues, and those with epilepsy had longer seizure-free intervals (27). However, evidence of effectiveness has been mixed, and uptake has been incomplete. A UK study indicated while cervical screening status was more likely to be recorded, over 65% of those with health checks did not obtain Pap smears (28). A 2019 Canadian study indicated that rates of preventative screening were not significantly different among those who did and did not use the health check tool (29). Health checks have been observed to result in slightly fewer preventable emergency room visits, although there was no observed effect on total emergency room visits, hospitalizations (27) or healthcare costs (30). Further, there have been issues with implementation of health checks in multiple countries. Physicians have expressed concern that the checks are time consuming and have noted a lack of evidence base for implementation (31). Although Australia and the UK implemented additional payments for completion (32, 33), this incentive has not had the desired impact: uptake rate has lagged below 60% in the UK (27) and well below 50% in Australia (33). Further, a study in the UK indicated that initiated health checks were completed in only 55% of patients studied (32).

### Health Passports

Hand-held health records, more commonly known as “health passports” have evolved as another method of communicating health information for those with IDD. Originally developed in Australia as a health “diary” to help people with IDD organize what they would like to communicate to their providers (34), passports have evolved into a hybrid of health history communication and healthcare preference guide to assure person-centered care. The passport is designed for use at the point of care. More than 60 versions have been developed, and, like health checks, they vary widely in format and length (35). Many passports prioritize information the user would like to share with their provider about their care preferences and indicate the user's communication needs. A few are formatted to meet the specific diagnostic information needs of the receiving provider (36). Passports have been more widely implemented in Australia and the UK than the US, and Canada is now evaluating their use. There is evidence that these patient-generated hand-held records are useful communication tools. Surveys of passport users have noted that its use improved communication with their providers (37) and in emergency departments, it minimized their need to answer the same questions for multiple providers (38). Passports were among the only outpatient information source available in the early days of the COVID 19 pandemic, when persons with IDD were quarantined in hospitals without their usual support staff (36). However, in two randomized, controlled studies, there was no evidence of improvement in health service delivery (37, 39). Multiple studies have indicated that the passport was not used consistently by providers, either because relevant information was not readily accessible or because they felt the information was already available to them from other sources (35, 38). In addition, caregivers and individuals have neither completed nor brought the passport to healthcare encounters consistently. One study indicated it was used by fewer than 40% of subjects (37). This mixed experience has led to calls for standardization and more rigorous testing of passports to improve future implementation (35, 40).

### Framework for Useful Information Tool Development

While poor information transfer is a well-documented barrier to health equity in the IDD population, the low uptake and mixed effectiveness of the current solutions suggest that it would be helpful to take a new approach. Recent methods of person-centered clinical tool development, such as Stanford-based Design Thinking (41) and Applied Systems thinking (42), have shown promise in improving the implementation of clinical practices. These design methods can provide valuable insights to designing more effective information tools; they not only engage users in the creation of the tools, but actively assess each user's needs, approach, and barriers to optimal performance during the iterative design process. In the case of information tool development, this is particularly helpful because each tool has two sets of users whose needs must be met: those who enter and those who receive the tool's information. With this design approach and the lessons learned from Health Checks and Passports in mind, we suggest the following guidelines for tool development that will retain the benefits but transcend the limitations of these tools (Table 1).

**Table 1:** Benefits and limitations of current health information transfer tools.

**Modality**	**Benefits**	**Limitations**
Healthchecks (Health Assessment Checklists)	• Detect health issues in IDD patients • Improve uptake of some preventative care • Decrease preventable emergency department visits • Improve documentation of chronic mobility and sensory issues • Improve epilepsy care	• For annual use only • No standard content • No standard data acquisition method • Inconsistent uptake by providers • No effect on hospitalization rate • No improvement in cost of care • Time consuming • Lack of standardization
Health Passports (Hand-held Health records)	• Prioritizes communication needs of patient • Indicates patient priorities • Perceived improved communication with providers	• RCTs indicate that there was no improvement in health service delivery • Information does not improve workflow • Low uptake by patients • Inconsistent or incomplete use • Inconsistent updates • Lack of standardization

#### Define the Communication Goal and Identify the Users

The European and Canadian experiences suggest that no one tool or document can meet the information needs of all parties in all contexts of all healthcare interactions. For example, a document formatted to communicate the information a provider needs for diagnosis and treatment may need to be separate from a tool that communicates an individual's personal interaction needs and preferences for care. Information important for optimal nursing care may not contain information relevant to physicians.

#### Create Separate Interfaces for Data Input and Data Output

The presentation of a filled-in data-acquisition form, where useful information is not highlighted and irrelevant information is not removed, risks burying relevant information and is less likely to be accepted by patient or provider (43). Tailoring the data interface to meet the needs of those entering information is more likely to improve the completeness and accuracy of the data. In addition, the data will be best received by placement into a thoughtful “reporting” format that respects the information needs of healthcare providers and each stakeholder involved in care. Ideally, information should be communicated concisely, in the order that the reader requires it.

#### Optimize the Simplicity and Accuracy of Data Entry

Because persons with IDD and their caregivers provide the key information base for creating a communication tool, care must be taken to be sure they can provide accurate, up-to date information. This can be challenging, as it is not uncommon for direct service providers to change frequently (44). Therefore, a standard process of data entry should be implemented that can be easily adopted with brief training, requires standard periodic updating (e.g. monthly or quarterly), and includes a method of monitoring for accuracy and data entry completion. If the tool is electronically based, monitoring can be done in a central.

#### Optimize the Integration Into the Workflow of all Stakeholders

The needs of patients with IDD do not suit the usual workflow of a healthcare encounter. The requirement of additional information not easily obtained during the usual healthcare encounter, as well as the difficulty detecting complex and unusual health needs in a population that comprises <3% of the patient base, cause disruptions that tax the allotted time and expertise of the provider. This causes the provider to care for the patient using inconsistent processes, which then risk errors and omissions. Information tools should therefore be focused on repairing the workflow disruptions by supplying all necessary information in a just-in-time fashion, like that expected from a neurotypical patient, and ideally, should give the provider an analysis of that information to assist in the assessment of a complex, unusual patient.

#### Meet Criteria of Quality

Once developed, each tool should be tested to meet predefined indicators of successful implementation. We recommend that information transfer tools designed for healthcare encounters have affirmative answers to seven questions that indicate effectiveness. They are: (1) Is it used consistently by caregivers and providers? (2) Does it improve evaluation speed? (3) Does it improve the correctness and timeliness of diagnosis? (4) Does it motivate correct clinical interventions? (5) Does it result in positive health outcomes? (6) Does it improve the patient's perception of care? and (7) Does it result in reduction of unmet health needs? Those tools that do not yield “Yes” answers in pilot testing should be reevaluated and iteratively improved before they are recommended for general use.

## Discussion

Creating effective communication tools useful in repairing communication failures for those with IDD is challenging, especially for use in the variable and fragmented U.S healthcare delivery system. It requires innovative strategies for acquiring, processing, and conveying relevant information. A design strategy that takes in to account the needs [e.g., jobs, pains and gains (45)] of each stakeholder is more likely to improve usage rates than those experienced in Europe, Australia, and Canada with the first iteration of these tools. Of note, the European experience has demonstrated that financial incentives will not induce healthcare providers to disrupt their workflow with inefficient communication methods. The communication tool also must be user friendly for caregivers and direct service providers who need to easily populate the tool regularly with accurate information. One U.S. based nonprofit organization, the Right Care Now Project (www.rightcarenowproject.org), has convened an interdisciplinary collaborative of information technology experts, implementation scientists, healthcare providers, service providers, advocates, and individuals with IDD to help develop a new generation of state-of-the art information transfer tools that are usable and actionable for all stakeholders, take advantage of advances in health record and other information technology, and build upon the healthcare improvements of health checks and passports (Table 2) (46). The team has developed a data repository that accepts information from caregivers, family, electronic health records, pharmacies and administrative datasets. Caregivers can easily access the system through a web-based interface (or paper questionnaire if necessary) and are guided to answer specific questions. The data is processed through algorithms designed to detect care omissions or active problems, and multiple person-centered and stakeholder-specific reports are generated to meet the information needs of each care interaction. These reports include tools to remind caregivers to promptly obtain necessary care, and tools that give an “at-a-glance” for the healthcare provider regarding healthcare needs, preferences, and important evidence of health, function, and quality-of-life changes over time. Importantly, the provider communication tool also puts the data into context with guidance statements that suggest important next steps in care, so the patient can be assured that all potential issues are addressed. By making sure the tools are formatted to include only relevant information in an expected order, the user does not need to struggle through a long data entry form and screen out irrelevant information. Most importantly, as an electronically based communication tool, this approach offers many advantages. First, through interoperability with electronic health records, it can bring evidence of a problem or an overdue preventative study to the attention of a healthcare provider far sooner than a scheduled appointment. Second, the data can be processed to evaluate for changes over time that indicate potential health problems that may otherwise go unnoticed until a crisis, and third, the data can be aggregated to detect and report population-level health issues to residential service providers or other administrative entities that may indicate a quality problem on a system level. In this way, one communication tool can be customized to meet the needs of each stakeholder in the person's Circle of Care (47).

**Table 2:** Potential features of next-generation information transfer tools.

•Combining patient-driven data input with checklist method of health issue detection to automatically detect health risk patterns
•Standardizing regular (e.g. monthly or quarterly), relevant data input from patient data sources
•Checking data for completeness, timeliness, and accuracy
•Communicating relevant information in a role-specific format to enhance workflow (e.g. Caregiver, case manager and healthcare provider-specific reports)
•Providing next-step guidance in a role-specific format to enhance workflow (e.g. Caregiver, case manager and healthcare provider-specific guidance and alerts)
•Detecting changes in symptoms and signs over time
•Monitoring for use of preventative care services
•Accepting electronic data into secure cloud
•Providing searchability for all data-input encounters and role-specific alerts
•Interoperability with all electronic health records

Given current advances in information technology, the promise of a communication-assisting tool that will improve health equity for those with IDD is within reach. By designing such tools with user-centered approaches and a clear focus on meeting the useability goals of simplicity, data accuracy, easy workflow integration, and positive outcomes, it may be possible to motivate adoption among all care stakeholders and bring important innovation into clinical practice for the IDD community.

## Data Availability Statement

The original contributions presented in the study are included in the article/supplementary material, further inquiries can be directed to the corresponding author.

## Author Contributions

PD and SA wrote the manuscript, designed and created the tables, assisted with manuscript drafting and approved the final manuscript. All authors contributed to the article and approved the submitted version.

## Funding

This research was funded by generous public contributions to the Right Care Now Project, Inc., a 501(c)3 nonprofit organization.

## Conflict of Interest

PD and SA were employed by the Right Care Now Project, Inc. Westborough, MA. SA is the cofounder and CEO of Advasys, Inc., a company that is developing technologies to improve the health and care of persons with developmental and intellectual disabilities.

## Publisher's Note

All claims expressed in this article are solely those of the authors and do not necessarily represent those of their affiliated organizations, or those of the publisher, the editors and the reviewers. Any product that may be evaluated in this article, or claim that may be made by its manufacturer, is not guaranteed or endorsed by the publisher.
